# Fatigue Life Assessment of API Steel Grade X65 Pipeline Using a Modified Basquin Parameter of the Magnetic Flux Leakage Signal

**DOI:** 10.3390/ma16020464

**Published:** 2023-01-04

**Authors:** Syed Muhamad Firdaus, Azli Arifin, Shahrum Abdullah, Salvinder Singh Karam Singh, Noorsuhada Md Nor

**Affiliations:** 1 Department of Mechanical and Manufacturing Engineering, Faculty of Engineering and Built Environment, Universiti Kebangsaan Malaysia, UKM, Bangi 43600, Selangor, Malaysia; 2 School of Civil Engineering, College of Engineering, Universiti Teknologi MARA, Cawangan Pulau Pinang, Permatang Pauh 13500, Pulau Pinang, Malaysia

**Keywords:** magnetic flux leakage, fatigue life, modified Basquin equation, reliability analysis, uniaxial cyclic test

## Abstract

This paper presents a modified fatigue life model of the Basquin equation using the stress parameter of the magnetic flux leakage signal. Most pipeline steels experience cyclic loading during service and the influence of the load history makes assessing fatigue behaviour more difficult. The magnetic flux leakage signal’s response to a uniaxial cyclic test of API X65 steel was measured with eight levels of ultimate tensile stress loads. The influence of dH(y)/dx on fatigue failure was the main concern in this study, the aim being to represent localised stress parameters in the modified Basquin equation. Both fatigue lives, experimental and predicted from the modified Basquin equation, were validated through reliability analysis, producing a 60% value when approaching 1.8 × 10^5^ cycles. The fatigue data from the experiment produced a higher mean-cycle-to-failure value than the prediction data, with slightly different values of 3.37 × 10^5^ and 3.28 × 10^5^. Additionally, the modified Basquin equation’s predicted and the experimental fatigue lives were found to have a high *R*^2^ correlation value of 0.9022. The Pearson correlation also showed a good relationship between the fatigue lives, with an *r* value of 0.9801. Finally, the modified Basquin equation based on dH(y)/dx signals provided an accurate and alternative method for durability assessment.

## 1. Introduction

The main consideration with the existing enormous pipeline structures is not the expansion of new materials or connecting techniques but their age. These outdated systems must keep operating securely and efficiently for many more years to meet the ongoing demand for oil and gas [1,2]. Failures are attributed to environmental factors, loading in design, construction, or operation. In addition, changes in operational frequency, temperature, and repetitive loads are potential sources of cracks that may eventually lead to simple failures [3]. Accurate analysis of a variety of long-lasting infrastructures, including pipelines, highways, and bridges, is crucial to lowering the exposure to system failure. Moreover, to offset the possible failure induced by fatigue, fracture studies appear to have been useful. The stress control approach has gained greater attention among those aiming to identify the residual life of engineering components and structures [4]. The Woehler (S-N)-curves, which depict the relationship between the applied load and a material’s fatigue life, were named after August Woehler, one of the field’s founders [5]. Mechanical stress–strain hysteresis studies are commonly used to investigate the cyclic deformation mechanism of metallic materials, but it has also become more common over the past two decades to use temperature, electrical resistance, and magnetic measurements [6,7]. It is necessary to generate S-N data that is suitable for the configuration of components and structures, in addition to selecting the best material. In some circumstances, it is necessary to extract samples from operational structures for additional testing due to the influence of ageing on the structures or components. As a result, the amount of material that may be used is significantly reduced, which is critical, given that the required full dataset must be collected using a minimal amount of material and at minimal time and expense. Until now, tradition and the ability to convert loads into stresses and strains have been the main reasons for utilising S-N curves with the structural fatigue configuration.

Engineers commonly employ non-destructive testing (NDT) to check for deficiencies in their work. One widespread and traditional type of NDT for damage detection is visual inspection [8]. Other NDTs can be used for early-stage damage inspection, including acoustic emission, eddy current, ultrasonic testing, radiography, and electromagnetic acoustic transducers. Defects with components, structures, or systems—especially pipelines—can be assessed by evaluating their properties without changing or damaging their original condition. However, these traditional NDTs only effectively reveal macro cracks in the structure, rather than predicting early damage. Engineering practice has widely embraced magnetic NDT technologies to provide operational security for ferromagnetic structures and components. Numerous non-destructive magnetic techniques, including stress-induced magnetic anisotropy (SMA), magnetic flux leakage (MFL), magnetic Barkhausen noise (MBN), magnetoacoustic emission (MAE), and the newly created metal magnetic memory (MMM) technique, have been developed during recent decades [9,10]. This is the result of using the physical basis of the piezomagnetic effect to evaluate the stress status of ferromagnetic components and structures by magnetic measuring methods compared with magnetostriction [11]. In the twenty-first century, the MMM technique is regarded as a new non-destructive testing methodology. It can provide an early diagnosis of ferromagnetic material and structures, in addition to locating problems [11,12]. The MMM technique is a passive magnetic testing method that differs from the conventional magnetic flux leakage method since it utilises the spontaneous surface micro magnetic signals of ferromagnetic materials to accurately identify the location and level of a stress concentration or anomaly [13,14,15]. The MMM technique employs a self-magnetisation leakage field (SMLF), which is exhibited in ferromagnetic and paramagnetic materials at high-density dislocations [16].

NDT in engineering analysis for the evaluation of related variables is the first step in that direction. If proper sensing techniques are adopted, the link between the development of non-linear damage growth and the numerous parameters instantaneously recorded by NDT techniques may offer more suitable ways to distinguish fatigue processes than the traditional stress-strain assessment [17,18]. The application of various NDT-associated techniques to determine local material conditions under cyclic loading yields more precise data, such as the behaviour of cyclic distortion in the region of very low plastic strains, low stresses, and several cycles to failure [19]. Fatigue failure occurs when ferromagnetic components undergo plastic deformation. However, areas of stress concentration due to the existence of micro-cracks caused by stress action could be detected at an early stage using macroscopic evaluation. Meng et al. characterised the fatigue damage of bovine compact bone using NDT [20]. Taheri et al. detected the stress corrosion cracking of a pipeline using an advanced eddy current NDT method [21]. Sahraei et al. and Shen et al. used the non-destructive magnetic flux leakage method to characterize the mechanical properties of steel subjected to a variety of heat treatment profiles [22,23]. Thus, a new objective is to use the MMM technique as a form of NDT due to its ability to detect and monitor initial defects in addition to identifying stress concentration locations caused by non-uniform metal structures and predicting the fatigue life of ferromagnetic components. An alternative method of linking the magnetics of ferromagnetic components with fatigue life was constructed to investigate the development potential of uniaxial fatigue life monitoring using a MMM method. The effect of more complex uniaxial loading is a particularly important influence on the fatigue life of materials compared to monotonic study. Hence the need to carry out fatigue life studies at different types of uniaxial stresses to establish more varied correlation relationships and confirm the capability of the magnetic flux leakage signal method in predicting the fatigue life of ferromagnetic metals [24,25].

This study aimed to assess the applied stress of the Basquin equation fatigue life prediction by considering the magnetic flux leakage stress parameter. This study applied fatigue life prediction based on an adaptation of the localised stress of the Basquin equation. The hypothesis of this study is as follows: if fatigue data can be evaluated using reliability analysis, the modified fatigue life model of the Basquin equation is appropriate. This study makes a substantial contribution to the understanding of the analogy between the magnetic flux leakage in fatigue life assessments and the local stress parameter of the Basquin equation.

## 2. Materials and Methods

**Figure 1:**
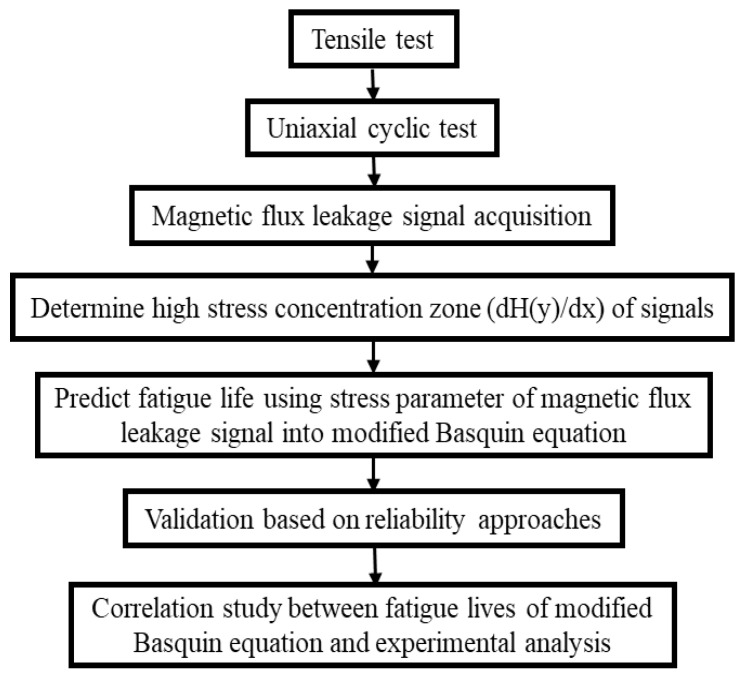
Methodology flow for modified fatigue life model of the Basquin equation process.

The methodology for this investigation is described in detail in this section. The methodology flow for this investigation is depicted in Figure 1. The methodology included a uniaxial fatigue test of API X65 steel, the magnetic flux leakage data from the fatigue test, the determination of high-stress concentration zones to identify the location on a specimen, fatigue life prediction using the Basquin equation based on the magnetic flux leakage parameter, validation of the predicted fatigue life using probabilistic approaches, and correlation analysis of the fatigue lives.

### 2.1. Tensile and Cyclic Test

**Figure 2:**
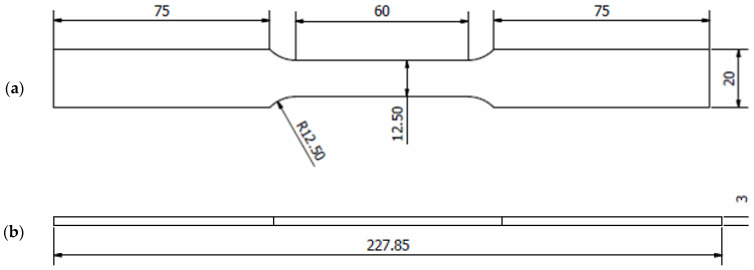
Details of the geometry of the API steel X65 specimens (unit in mm); (**a**) upper geometry schematic and (**b**) side geometry schematic.

**Figure 3:**
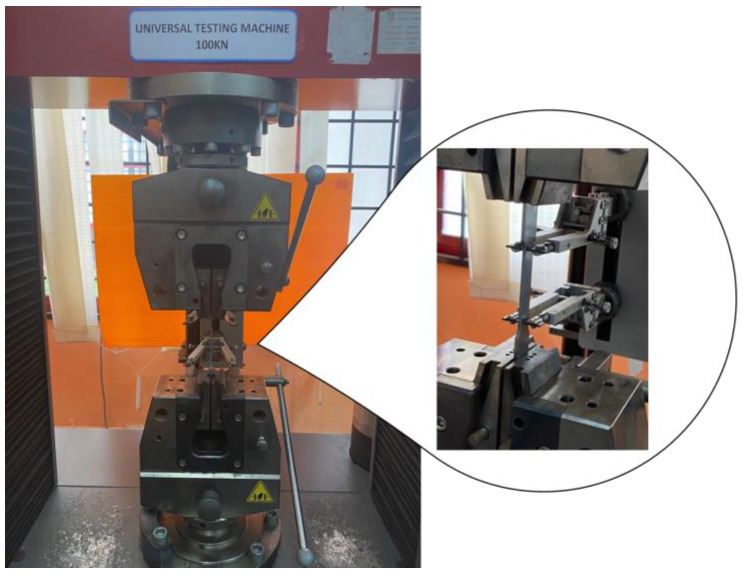
Specimen set-up for tensile test.

The material utilised in this study was ferromagnetic steel grade X65, which has been extensively used in the oil and gas sectors [1,26,27]. The chemical properties (wt%) of the API X65 steel studied are given in Table 1. To determine their monotonic properties, specimens with lengths of 227.85 mm underwent testing corresponding to the ASTM-E8 standard, as illustrated in Figure 2. The tensile test was conducted using a 100 kN universal testing machine at room temperature, as shown in Figure 3. The results provided detailed information about the strength and ductility of the material. In the tensile test, the strain rate was adjusted to 1 × 10^−3^ s^−1^. According to ASM International, the range of strain rates used for tensile tests should be 10^−5^ s^−1^ to 10^−2^ s^−1^. High strain rates ranging from 10^−1^ s^−1^ to 10^1^ s^−1^ can cause changes in the temperature and behaviour of a material [28].

**Figure 4:**
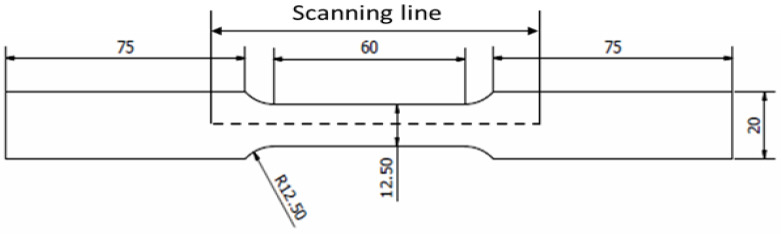
API X65 specimen geometry with scanning line (unit in mm).

**Figure 5:**
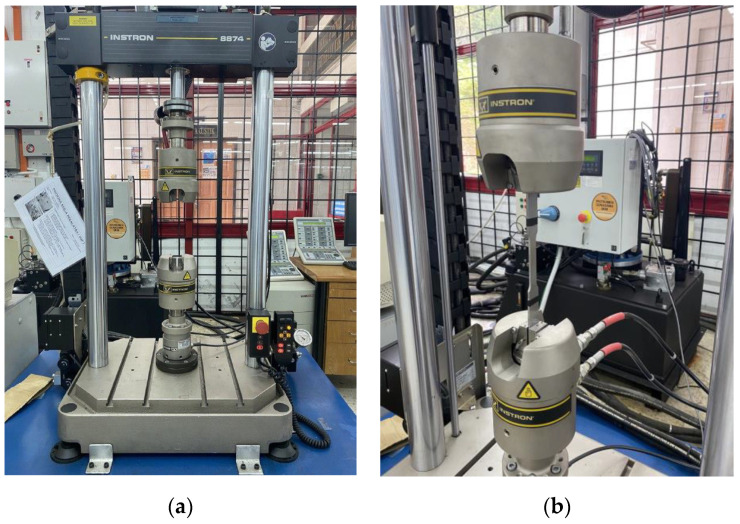
Specimen set-up for the cyclic test; (**a**) 25 kN servo-hydraulic machine, (**b**) ASTM E466 standard specimen equipped onto jigs of the machine.

**Figure 6:**
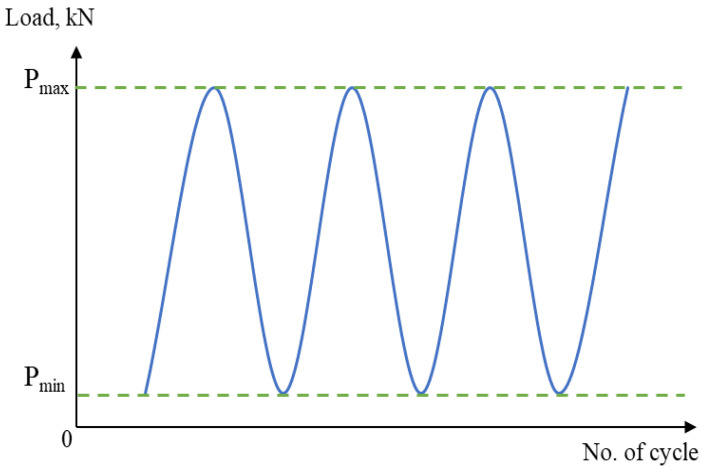
Constant amplitude loading schematic design for the cyclic test.

In addition, cyclic loading, specifically for dynamically loaded structures, has a crucial influence on service safety and reliability [29,30]. Therefore, to obtain the fatigue behaviour of the material, a cyclic uniaxial fatigue test was carried out using a 25 kN servo-hydraulic machine in line with ASTM E466, as shown in Figure 4 and Figure 5. Based on ASTM standard E466, at least eight specimens should be used to form a complete S-N curve. The applied load was also varied, based on the final value of the tensile strength, and the load value was reduced until the specimen yielded a value cycle above 10^7^ cycles, which is the fatigue limit (also known as the endurance limit) of a material [31]. Each specimen was subjected to uniaxial fatigue testing at a frequency of 10 Hz and with loads ranging from 50% to 85% of the Ultimate Tensile Strength (UTS) of API steel grade X65, which was obtained from the tensile testing, to ascertain how load variations affected the magnetic signals. The selection of the frequency value was based on the frequency used in previous studies that used the metal magnetic memory method during fatigue testing of metal materials [28,32,33]. In addition, Arifin et al. found that the fatigue test was unstable when using a frequency of 20 Hz during testing [34]. The maximum forces, P_max_, and the minimum forces, P_min_, were proportionate to the UTS load percentages, whereas the stress ratio R was set to 0.1, as illustrated in Figure 6 and Table 2.

### 2.2. The Acquisition of Magnetic Flux Leakage Data

**Figure 7:**
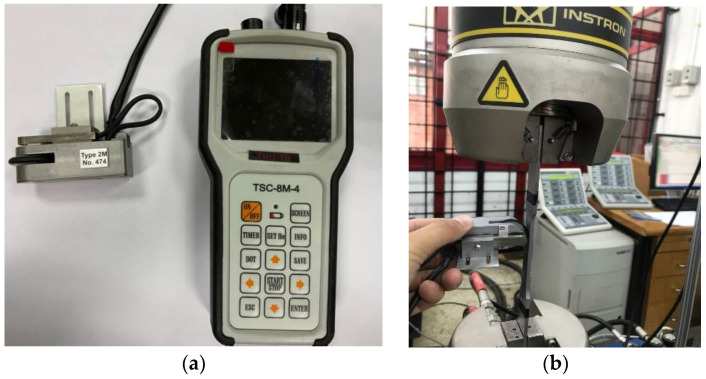
Image of data acquisition for magnetic flux leakage signals; (**a**) Stress concentration testing device for the identification of magnetic flux leakage signals, (**b**) Signal acquisition activity during a cyclic test.

An MMM scanning device, as shown in Figure 7a, was used to scan the magnetic flux leakage response data as part of the uniaxial fatigue test method. The lift-off rate was set to 0 mm, and the wheel centre’s separation from the sensor was set at 5 mm. In this study, the distance mode was used to measure the magnetic flux leakage signal response. The data was then collected by rolling the MMM scanning device along an 80 mm scanning line because this area was within the projected range of failure occurrence, as illustrated in Figure 7b. The cyclic test was performed on the specimen until it failed. MMM data acquisition occurred every 2000 cycles until the specimen failed. Following a similar experimental procedure, the remaining load was repeated with the other seven specimens at different loads.

### 2.3. Determination of Signals from High Stress Concentration Zones

The MMM method is based on applying a strong magnetic field to magnetise a ferrous metal object to the point of saturation. The MMM technique applies a combination of a geomagnetic field and mechanical stress due to defects and stress, utilising the spontaneous magnetisation induced by ferromagnetic materials. The magnetic flux is not disrupted if the object is free of abnormalities. The magnetic flux leakage component, H(y), which is perpendicular to the surface, changes its polarity when stress concentration zones are detected and its gradient, dH(y)/dx, reaches a maximum value. These are the two main components of the MMM method [35]. Equation (1) was used to illustrate the magneto-mechanical effect on a ferromagnetic material, which corresponds to an associated magnetic field in a geomagnetic field [36]:

(1) $$H_{\sigma }=\frac{3}{2}\frac{\sigma }{\mu_{0}}{\left(\frac{{d\lambda }_{s}}{\mathrm{dM}}\right)}_{\sigma }\left(\cos^{2}{\theta -\nu \;\mathrm{vsin}}^{2}\theta \right)$$

where σ is the applied stress, μ_0_ is the vacuum permeability, λ_s_ is the magnetostriction coefficient, M is the magnetisation, θ is the angle between the stress direction and magnetisation direction, and ν is the Poisson ratio. Thus, the magnetic leakage field induced by the applied stress is indicated by H(y). With increases in loading cycles, the stress of specimens varied continuously, while the H(y) associated with the induced magnetic field also changed repeatedly [32,36].

The gradient component of the obtained raw signals, dH(y)/dx, was created by converting the normal component of the magnetic intensity, H(y), to the acquired raw signals, as presented in Equation (2):

(2) $$\frac{{\mathrm{dH}}_{p}\left(y\right)}{\mathrm{dX}}=\frac{{\Delta H}_{p}\left(y\right)}{\Delta X}$$

This made it possible for the method to provide the best assessment of damage severity. [37]. The dH(y)/dx signals were chosen for additional analysis to identify the severity of the flaws on the surface of the scanned specimen [34].

### 2.4. Stress Parameter Adaptation in the Basquin Equation for Fatigue Life Prediction

An S-N curve is a plot that illustrates stresses against the number of cycles that a material can accommodate. This stress-based approach considers that local stress is the parameter controlling the fatigue life. The relationship between stress amplitude and the fatigue life or S-N curve can be represented using the Basquin equation, as shown in Equation (3) [38]:

(3) $$S_{a}{=\mathrm{AN}}_{f}{}^{B}$$

where S_a_ is the applied alternating stress, N_f_ is the fatigue life cycle, and A and B are the material coefficients.

The fatigue life obtained from the fatigue experiment was correlated with the magnetic flux signal recorded from the beginning of the experiment until fracture. In this study, the dH(y)/dx parameter was substituted into the Basquin equation to replace the stress amplitude parameter as the former reconsidered the equivalent parameters due to its potential to indicate localised stresses and their severity levels [39].

### 2.5. Fatigue Reliability Assessment

To estimate a component’s safety level under particular service conditions, fatigue reliability analysis computes the probability that the component would fail due to fatigue within a given service life range [40]. Fitting fatigue data into a probability density function (PDF) is the first stage when investigating fatigue reliability. Fatigue data can be represented by the Weibull distribution, according to earlier studies [41]. Equation (4) contains the base 10 logarithmic formula for the PDF of the fatigue life–Weibull distribution [42]:

(4) $$f_{\mathrm{weibull}}\left(N_{f}\right)=\frac{\beta }{\theta }{\left(\frac{N_{f}}{\theta }\right)}^{\beta -1}e^{{\left(\frac{N_{f}}{\theta }\right)}^{\beta }}$$

where β is the shape parameter and θ is the scale parameter. Maximum likelihood estimation (MLE), a prominent technique for determining the ideal distributional parameters, was used to fit the fatigue data into the Weibull PDF [41].

The following information is provided for the cumulative density function (CDF) of the Weibull distribution, in which the cumulative failure probability of the components across operation cycles is expressed in Equation (5) [40].

(5) $$F\left(N_{f}\right)=\int_{-\infty }^{\infty }f\left(N_{f}\right){\mathrm{dN}}_{f}{=1-e}^{{-\theta N}_{f}{}^{\beta }}$$

Additionally, the CDF can be used to compute the dependability function in the approach, as shown in Equation (6) [40].

(6) $$R\left(N_{f}\right)=1-F\left(N_{f}\right){=e}^{-{\theta N}_{f}{}^{\beta }}$$

The ratio of the PDF to the reliability function, as shown in Equation (7), can be used to define the hazard rate or failure rate function [40].

(7) $${\lambda (N}_{f})=\frac{f\left(N_{f}\right)}{R\left(N_{f}\right)}{=\theta \beta N}_{f}{}^{\beta -1}$$

Mean-time-to-failure (MTTF) is commonly determined by a fatigue reliability study to statistically assess fatigue reliability [39,40,41]. Mean-cycle-to-failure (MCTF) was utilised in place of mean-time-to-failure (MTTF) since the anticipated fatigue life was calculated in cycles. The MCTF for pipeline steel is presented in Equation (8).

(8) $$\mathrm{MCTF}=\int_{0}^{\infty }N_{f}\cdot f\left(N_{f}\right)d\left(N_{f}\right)=\theta \cdot \Gamma \left(\frac{1}{\beta }+1\right)$$

The projected number of operation life cycles before the first failure occurs is often regarded as the MCTF.

## 3. Results and Discussion

This section discusses the data obtained from the uniaxial fatigue test, the magnetic flux leakage signal characterisation based on the UTS cyclic load, the fatigue life prediction from the adapted Basquin equation, the reliability based on probabilistic approaches, and the correlation analyses between the experimental and prediction fatigue lives.

### 3.1. Fatigue Reliability Assessment

**Figure 8:**
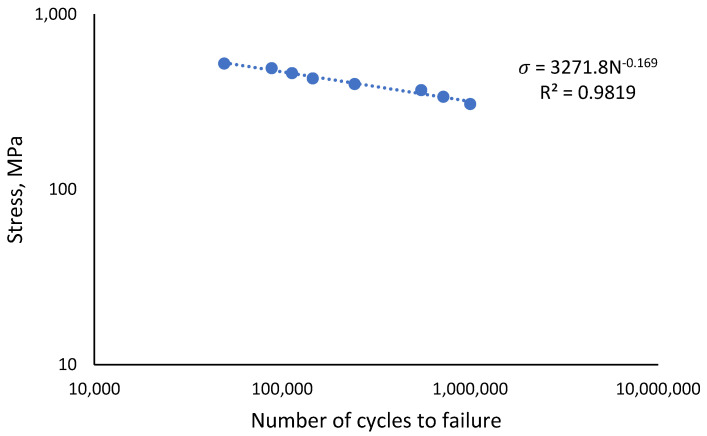
Fatigue behaviour of API steel X65.

The monotonic properties obtained from the tensile testing of API X65 steel are displayed in Table 3. In this work, the UTS value was used to design the load for the uniaxial cyclic testing. Its value of 614 MPa was divided into eight distinct types of loads, with percentages ranging from 50% to 85% of the UTS load, as shown in Table 3. The stress-life (S-N) curve was plotted using eight data points from the load percentage reductions, based on the UTS value. The curve shows the fatigue behaviour of the material achieved an endurance limit at 50% load, with a coefficient of determination value, *R*^2^ of 0.9819, as illustrated in Figure 8.

**Figure 9:**
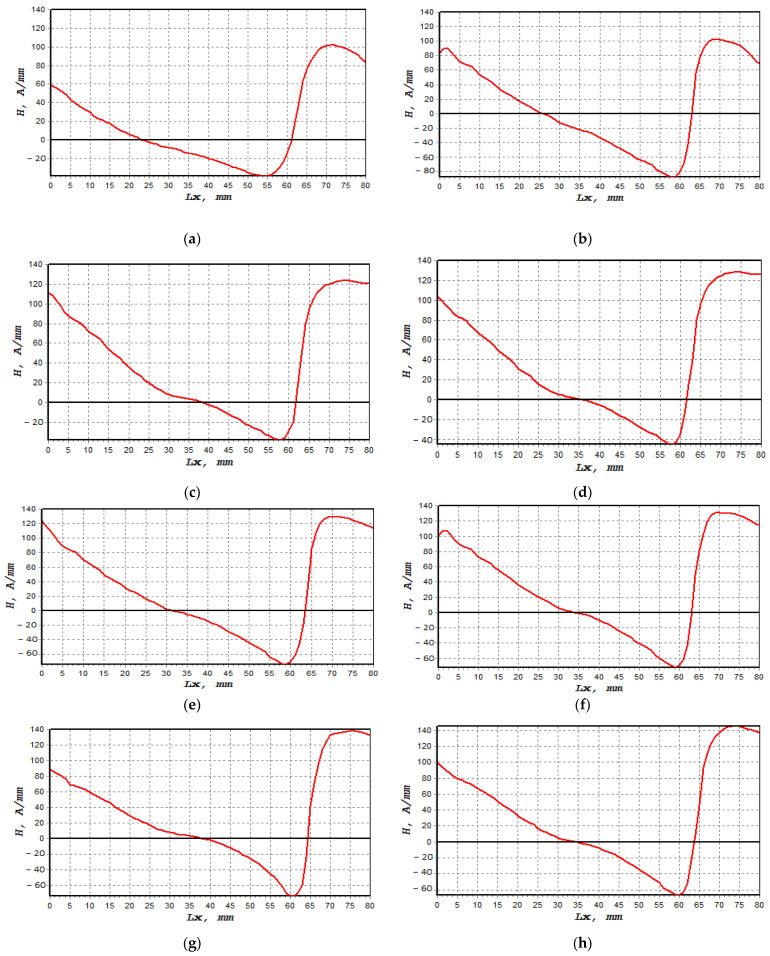
H(y) signal measurements based on UTS cyclic loading percentages: (**a**) 50%, (**b**) 55%, (**c**) 60%, (**d**) 65%, (**e**) 70%, (**f**) 75%, (**g**) 80% and (**h**) 85%.

**Figure 10:**
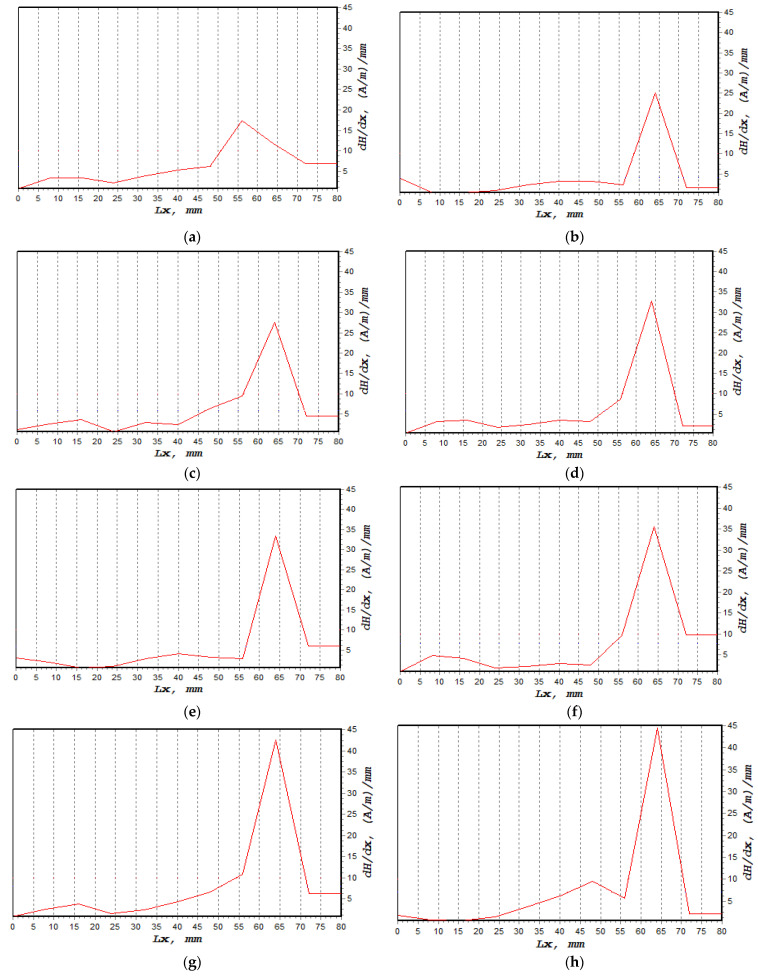

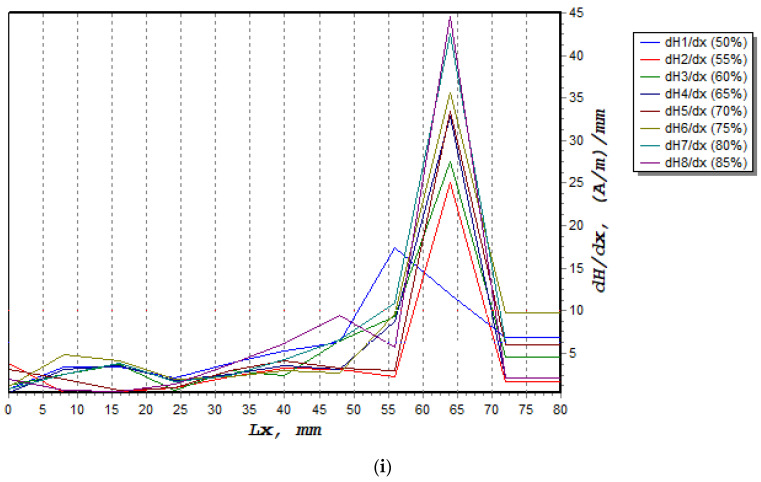
dH(y)/dx signal measurement based on UTS cyclic loading percentage; (**a**) 50%, (**b**) 55%, (**c**) 60%, (**d**) 65%, (**e**) 70%, (**f**) 75%, (**g**) 80%, (**h**) 85%, (**i**) combination of all dH(y)/dx signals.

The normal component of the magnetic intensity, H(y), showed a higher value at 85%, decreasing to 80% of UTS before it became constant between 75% and 50%, as shown in Figure 9. The magnetic intensity dropped constantly from 0 mm until the signal started to increase sharply at 60 mm. These behaviours, shown in Figure 9a–h, described abnormal activity in these regions when the magnetic intensity started to slightly decrease again at 70 mm. Table 4 presents the fatigue behaviour of the specimen associated with the applied loads, along with the magnetic flux reading. Figure 10a shows the lowest dH(y)/dx value with 19.4 (A/m)/mm, whereas the highest value was 44.5 (A/m)/mm, as depicted in Figure 10h,i illustrate the combination of magnetic flux signals’ response from 50–85% UTS loads. Furthermore, the recorded dH(y)/dx signals showed increasing amplitude values between 50% and 85% of the UTS load, reflecting how the load variation itself affected the magnetic flux leakage readings. Since the high-concentration zone was where the specimen had been damaged prior to failure, high concentrations of the dH(y)/dx values within these locations, from 50 mm to 75 mm, were projected. Due to irreversible alterations in the magnetic domain orientation brought on by the operational stress caused by the magnetic field, the magnetic field leakage developed at the stress concentration site [43].

### 3.2. Application of Magnetic Flux Leakage Stress Parameter into the Basquin Equation for Fatigue Life Prediction

**Figure 11:**
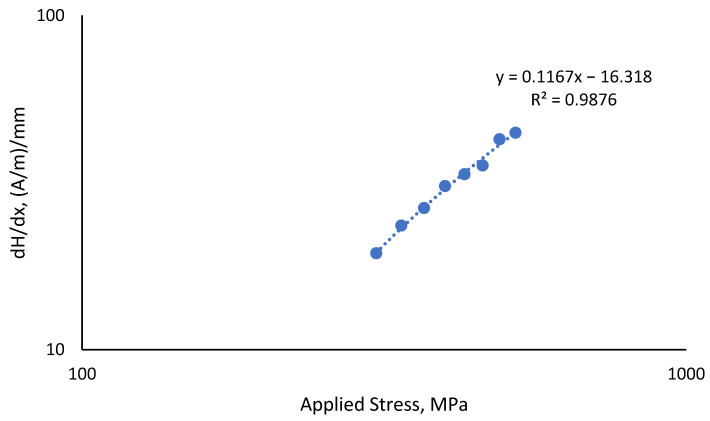
Correlation of dH(y)/dx value with all uniaxial applied stresses.

In this experiment, the dH(y)/dx ranged between 19.4 (A/m)/mm and 44.5 (A/m)/mm, as shown in more detail in Table 5. The stress value was directly proportional to the observed dH(y)/dx value, as shown in Figure 11, and both were inversely proportional to the fatigue life of the material, with the coefficient of determination value, *R*^2^, being 0.9876.

**Figure 12:**
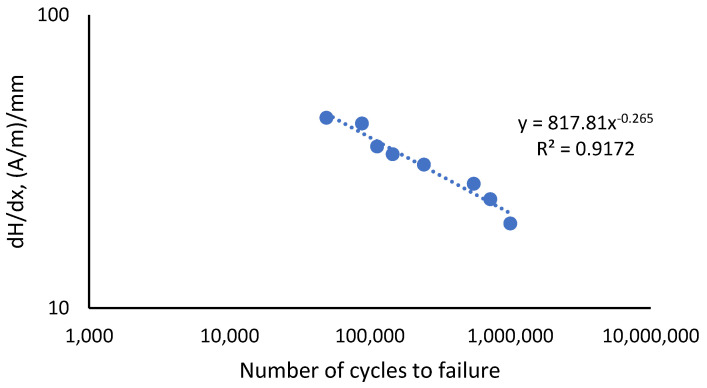
Correlation of dH(y)/dx value with all experimental fatigue life.

The correlation plot in Figure 12 shows the values of the coefficient of dH(y)/dx value against the fatigue life of the API steel grade X65 for all types of stresses. The dH(y)/dx value was seen to decrease with an increase in the fatigue life of the material when under the influence of increasing stresses, forming a straight line on the log scale. A straight line was obtained by fitting the curve with the value of the coefficient of determination, *R*^2^, which was 0.9172, indicating the plotted graph was within the acceptable range. The dH(y)/dx correlation was derived from the use of the Basquin equation, given in Equation (3), which was based on the stress-life curve equation. From the curve plot of the dH(y)/dx against the number of cycles, Equation (9) was proposed.

(9) $$\frac{\mathrm{dH}(y)}{\mathrm{dx}}=817.81{\left(N_{f}\right)}^{-0.265}$$

where dH(y)/dx is the value of the gradient of magnetic intensity, 817.81 is the coefficient, −0.265 is the exponent that meets the exponential range for the ductile material, and N_f_ is the fatigue life. Equation (9) is applicable to the specific case study presented in this paper. The predicted life using the adaptation of dH(y)/dx into the Basquin equation, Equation (9), is tabulated in Table 5. The comparison between the obtained fatigue lives from the Basquin equation and the modified Basquin model showed a small margin of percentage difference as shown in Table 5 with the highest difference of 11.3 % for 85 % UTS load.

### 3.3. Validation Based on Fatigue Reliability Analysis

**Figure 13:**
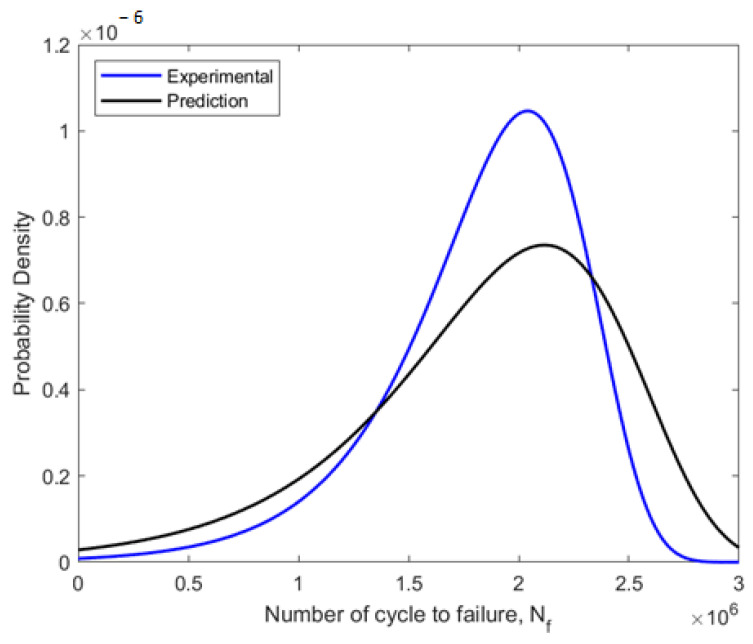
Weibull distribution PDF fitted using experimental and predicted fatigue data.

**Figure 14:**
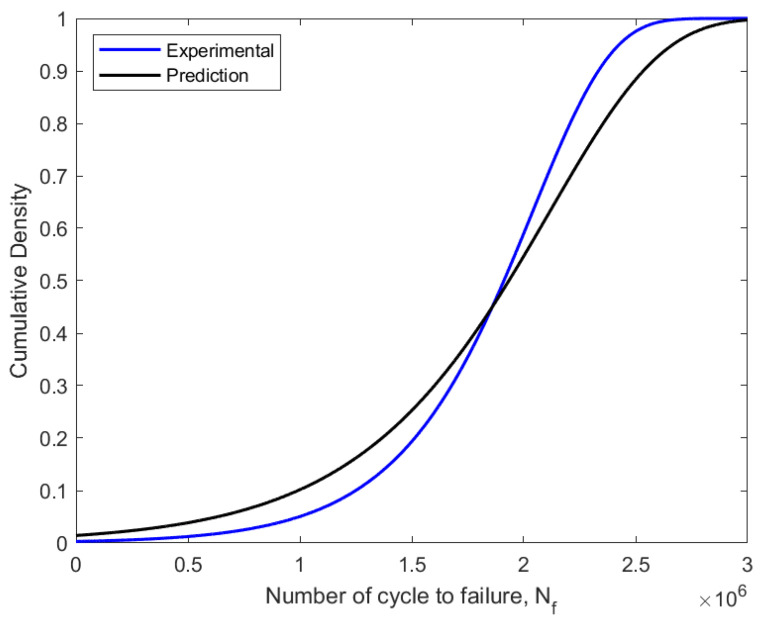
Weibull distribution CDF fitted using experimental and predicted data on fatigue.

**Figure 15:**
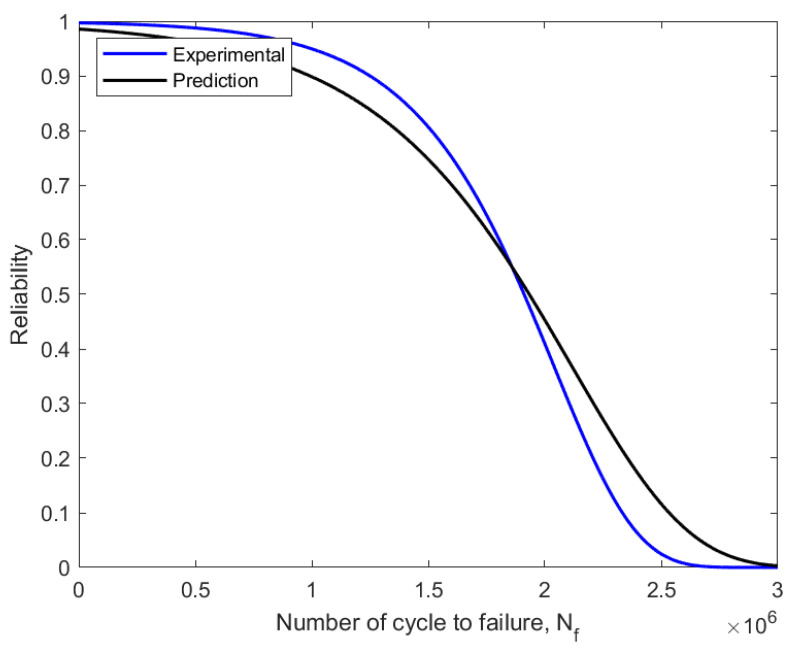
Reliability curve using experimental and predicted fatigue data.

**Figure 16:**
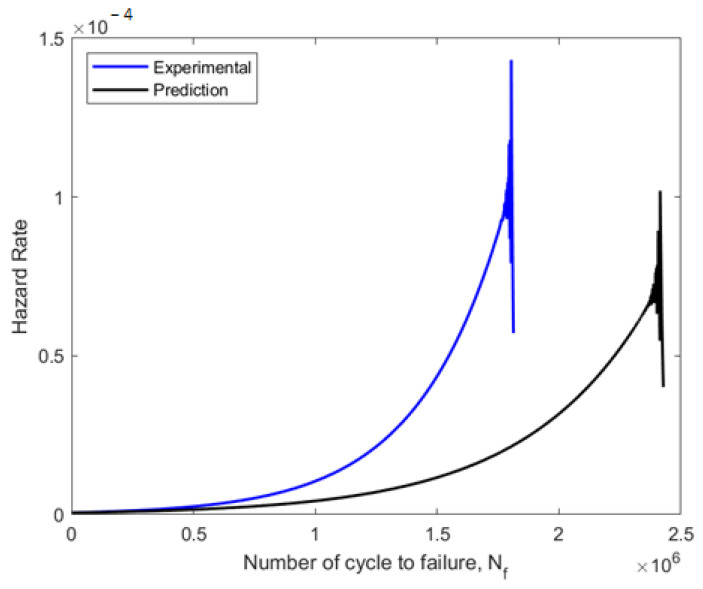
Weibull distribution for hazard rate curve for experimental and predicted data on fatigue.

Due to its capacity to estimate the fatigue life of components or structures, fatigue reliability evaluation is a crucial engineering strategy. Using the MLE approach to probabilistic analysis, the fatigue data were fitted into the Weibull distribution. The ideal Weibull parameters for the fitted fatigue data obtained using the MLE approach are shown in Table 6. The Weibull distribution-fitted PDF shown in Figure 13 used the experimental and predicted fatigue data. In regard to the fatigue life data, the PDF curves demonstrated that the probability of a component failure due to fatigue would fall between zero and a particular number of cycles. The prediction data were skewed to the right compared to experimental data, with experimental data showing the highest probability of failure at 1.1 × 10^−6^ compared to prediction with 0.7 × 10^−6^ value. These indicate that this pipeline failure allowed better fatigue life prediction with lower probabilities of failures. Then, using the estimated shape and scale parameters, the CDF, reliability function, and hazard rate function were calculated. The CDF, reliability function, and hazard rate function, which were computed from the fatigue life based on the experimental and predicted data, respectively, are shown in Figure 14, Figure 15 and Figure 16.

The failure probability of components or structures before a relationship with a time value was assessed using the CDF value in the assessment of fatigue reliability, which is frequently referred to as unsatisfactory [34]. The CDF curve also explained the connection between the failure probability and the service life prediction of fatigue. Thus, as the fatigue life cycle count, N_f_, rises from zero to one, the CDF value rises as well. The reliability function drops from one to zero with an increase in the number of fatigue life cycles. In the fatigue reliability assessment, the reliability values were represented as the percentage of the probability that a component or structure will survive. As a result, the pipeline steel service life was evaluated based on the applied loads and over a specific amount of time.

As shown in Figure 15, the definition of reliability assessment in engineering is the probability that a system or component will fail due to technical issues within a given time frame and under a specific set of operating conditions. Figure 15 shows the reliability function decreased to zero from the value of component survival before failure, with 0.6297 (62%) for the experimental model and 0.6180 (61%) for the prediction model. One component of dependability that has been studied such that reliability can be realistically modelled using statistical features is the failure probability. Additionally, based on the applied loading, fatigue reliability was used to assess a component’s capacity to withstand a specific number of cycles before failing [44].

The component failure probability was then determined using the hazard rate function after the component had operated for the specified cycles. The hazard rate plot frequently displayed a high rate of the first failure as the component age increased, followed by a duration of relative reliability, before a period of a rapid escalation in the rate. The hazard rate curves for the experimental data plot and the forecast data plot were compared, as shown in Figure 16. With shape parameters of more than 1, the experimental and predicted fatigue data showed growing failure rates. The experimental data illustrated a higher hazard rate of failure at 1.4 × 10^−4^ (1.8 × 10^6^ blocks to failure), compared to the prediction data hazard rate of 1.1 × 10^−4^ (2.4 × 10^6^ blocks to failure). As a result, it can be shown that both sets of fatigue data indicated a higher probability of fatigue failure at a later phase of the specimen’s operational life [42].

Table 7 depicts the MCTF of the fatigue data computed by the experimental uniaxial test and the prediction using the substitution of the dH(y)/dx parameter into the Basquin equation. The fatigue data from the experiment gave higher MCTF values than the prediction data, with the tiny margin being 3.37 × 10^5^ cycles and 3.28 × 10^5^ cycles. This clearly indicated that the dH(y)/dx parameter can be applied in the Basquin equation to estimate fatigue life through the equivalence of localised stress parameters. The Weibull probability curve was fitted with the fatigue data arising from the use of several durability models in this work to determine the utility of fitting the dH(y)/dx parameter adaptation into the Basquin equation to estimate fatigue life.

**Figure 17:**
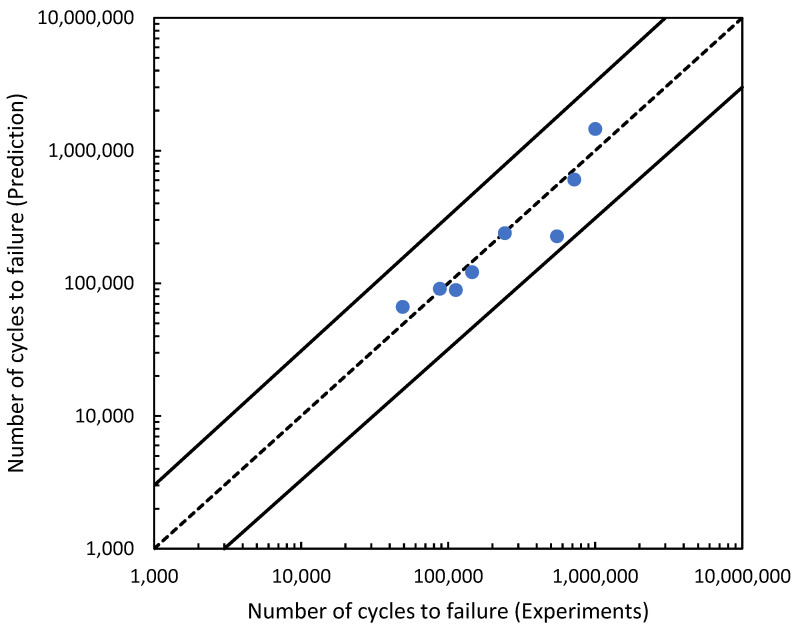
Correlation curve for the modified fatigue life model of the Basquin equation and experiments fatigue life.

**Figure 18:**
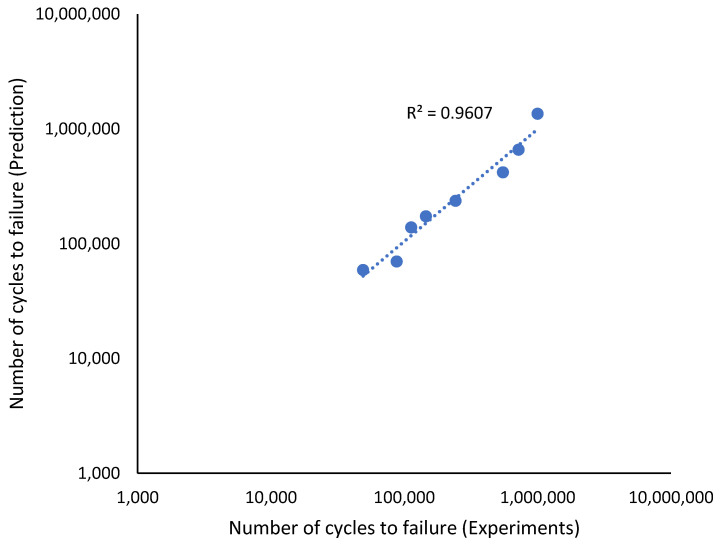
Relationship between the modified fatigue life model of the Basquin equation and experiments fatigue life.

**Figure 19:**
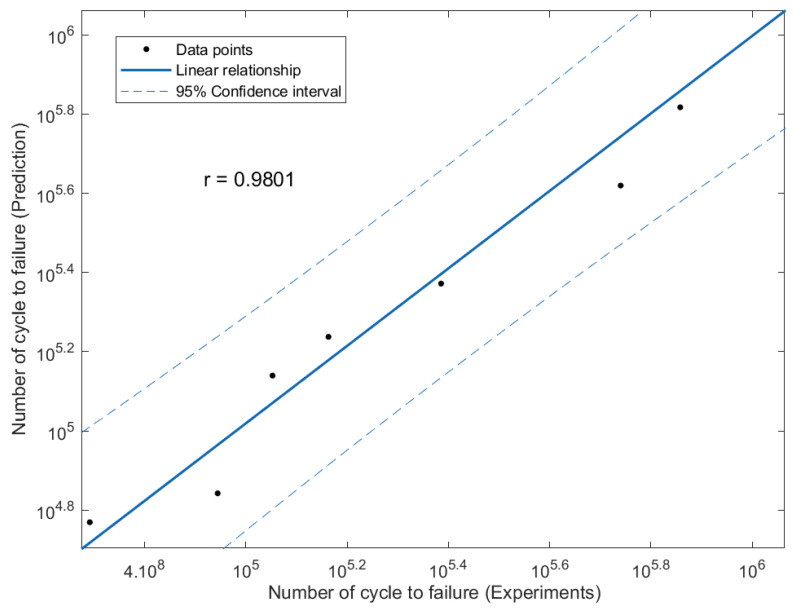
Linear regression for the modified fatigue life model of the Basquin equation and experiments fatigue life.

The effectiveness of the predictions of the fatigue life analysis was assessed using 1:2 or 2:1 correlation curves [45]. The correlation between the modified fatigue life model of the Basquin equation and the experimental fatigue life is shown in Figure 17. According to the correlation plot, all the fatigue life data points are within the 1:2 or 2:1 boundaries. Figure 18 shows how the linear regression method was used to further evaluate the link between the prediction and the experimental fatigue life. In Figure 18, the *R*^2^ value of the modified fatigue life model of the Basquin equation and the experimental fatigue life was 0.9156. A greater data fit between the regression and the data is shown by higher *R*^2^ values. In Figure 19, the Pearson correlation coefficient, *r*, between the fatigue lives was computed, producing a value of 0.9801. This strongly indicated that the fatigue life of the prediction model was directly proportional to the experimental fatigue life since the 95% confidence interval covered all the data points. The direction and magnitude of a linear relationship were displayed using the *r* value. Uncertainty or inaccuracy must compensate for the severity of the prediction’s significant value when it is inaccurate [46].

## 4. Conclusions

- The MMM approach was employed in this investigation to characterise the uniaxial fatigue loading, which ranged from 50% to 85% UTS loading, based on the magnetic flux leakage measurements. The results showed that 85% UTS load gave higher H(y) and dH(y)/dx values as a higher load affected the magnetic flux leakage reading. All the magnetic flux leakage signals indicated that regions of abnormalities were detected from 50 mm to 75 mm.
- The stress-life curve showed the fatigue behaviour of the API steel grad X65, which achieved an endurance limit of 307 MPa, approximated at 50% of the UTS cyclic load. The curve shows a good *R*^2^ correlation value of 0.9819 between the stress applied and the number of cycles to failure.
- In comparison to the magnetic flux leakage value, the dH(y)/dx obtained for the experimental fatigue life showed a good correlation analysis, with an *R*^2^ value of 0.9156 and a Pearson correlation value of 0.9801.
- The localised stress of the magnetic flux leakage and the fatigue life from the experimental model indicated that the parameter could substitute for the stress parameter of the Basquin equation. The MCTF value of the reliability assessment and the linear regression between these fatigue lives also indicated a good correlation, with slightly different values.

These findings indicate a strong justification for the substitution of the dH(y)/dx parameter into the Basquin equation. Therefore, the proposed modified fatigue life model of the Basquin equation using the dH(y)/dx parameter is recommended for fatigue life assessment.

## Figures and Tables

**Table 1:** The chemical properties of the API steel grade X65 [28].

Elements	C	Mn	Ni	Mo	Ti	Si	Cr	S	Fe
Weight	0.06	1.60	1.29	0.60	0.30	0.23	0.16	0.11	Balance

**Table 2:** Load design for the cyclic test based on UTS percentage.

Load (%)	P_min_ (kN)	P_max_ (kN)	Stress Ratio, R
50	1.9	19.5	0.1
55	1.8	18.4	0.1
60	1.7	17.2	0.1
65	1.6	16.1	0.1
70	1.5	14.9	0.1
75	1.3	13.8	0.1
80	1.2	12.6	0.1
85	1.1	11.5	0.1

**Table 3:** Monotonic properties of API steel X65.

Properties	Yield Strength, MPa	Ultimate Tensile Strength, MPa	Young’s Modulus, GPa
Values	572	614	220

**Table 4:** Characterisation of magnetic flux leakage behaviour during uniaxial fatigue testing.

UTS Load, %	Applied Stress, MPa	Experimental Number of Cycles to Failure, N_f_	dH(y)/dx, (A/m)/mm
50	307.0	1,002,150	19.4
55	337.7	721,273	23.5
60	368.4	550,000	26.5
65	399.1	243,067	30.8
70	429.8	145,608	33.4
75	460.5	112,868	35.5
80	491.2	87,952	42.5
85	521.9	49,158	44.5

**Table 5:** Fatigue lives of API steel grade X65.

UTS Load, %	dH(y)/dx, (A/m)/mm	Experimental Number of Cycles to Failure, N_f_	Prediction (Basquin) Number of Cycles to Failure, N_f_	Difference, %	Prediction (Modified Basquin) Number of Cycles to Failure, N_f_	Difference, %
50	19.4	1,002,150	1,204,369	10. 7	1,349,201	25.7
55	23.5	721,273	685,223	4.4	656,072	9.9
60	26.5	550,000	409,478	1.6	416,306	32.4
65	30.8	243,067	254,999	8.2	235,505	3.2
70	33.4	145,608	164,473	4.8	172,774	15.7
75	35.5	112,868	129,345	6.2	137,943	18.1
80	42.5	87,952	74,636	7.2	69,595	26.3
85	44.5	49,158	52,138	11.3	58,833	16.4

**Table 6:** Scale and shape parameter of the Weibull distribution.

Model	Weibull Parameter	
	Scale Parameter	Shape Parameter
Experimental	3.76 × 10^5^	1.0833
Prediction	3.89 × 10^5^	1.0153

**Table 7:** MCTF of fatigue data obtained by experiment and prediction.

Obtained Fatigue Life	Mean-Cycle-to-Failure, Cycles
Experimental	3.37 × 10^5^
Prediction	3.28 × 10^5^

## Data Availability

The processed material or data required to reproduce these findings cannot be shared at this time as the data also form part of an ongoing study.
